# Natural Products for the Treatment of *Chlamydiaceae* Infections

**DOI:** 10.3390/microorganisms4040039

**Published:** 2016-10-16

**Authors:** Mika A. Brown, Michael G. Potroz, Seoh-Wei Teh, Nam-Joon Cho

**Affiliations:** 1 School of Materials Science and Engineering, Nanyang Technological University, 50 Nanyang Avenue, Singapore 639798, Singapore; mika.a.brown@gmail.com (M.A.B.); mikepotroz@gmail.com (M.G.P.); seohwei1208@gmail.com (S.-W.T.); 2 Centre for Biomimetic Sensor Science, 50 Nanyang Drive, Singapore 637553, Singapore; 3 Department of Chemical Engineering, Northeastern University, 360 Huntington Avenue, Boston, MA 02115, USA

**Keywords:** *Chlamydiae*, *Chlamydiaceae*, *Chlamydia*, chlamydial infections, natural products, antibacterial

## Abstract

Due to the global prevalence of *Chlamydiae*, exploring studies of diverse antichlamydial compounds is important in the development of effective treatment strategies and global infectious disease management. *Chlamydiaceae* is the most widely known bacterial family of the *Chlamydiae* order. Among the species in the family *Chlamydiaceae*, *Chlamydia trachomatis* and *Chlamydia pneumoniae* cause common human diseases, while *Chlamydia abortus*, *Chlamydia psittaci*, and *Chlamydia suis* represent zoonotic threats or are endemic in human food sources. Although chlamydial infections are currently manageable in human populations, chlamydial infections in livestock are endemic and there is significant difficulty achieving effective treatment. To combat the spread of *Chlamydiaceae* in humans and other hosts, improved methods for treatment and prevention of infection are needed. There exist various studies exploring the potential of natural products for developing new antichlamydial treatment modalities. Polyphenolic compounds can inhibit chlamydial growth by membrane disruption, reestablishment of host cell apoptosis, or improving host immune system detection. Fatty acids, monoglycerides, and lipids can disrupt the cell membranes of infective chlamydial elementary bodies (EBs). Peptides can disrupt the cell membranes of chlamydial EBs, and transferrins can inhibit chlamydial EBs from attachment to and permeation through the membranes of host cells. Cellular metabolites and probiotic bacteria can inhibit chlamydial infection by modulating host immune responses and directly inhibiting chlamydial growth. Finally, early stage clinical trials indicate that polyherbal formulations can be effective in treating chlamydial infections. Herein, we review an important body of literature in the field of antichlamydial research.

## 1. Introduction

Effective management of infectious diseases is one of most important endeavors of modern times. Infectious diseases impact human, animal, and environmental health, and beyond the direct cost of human disease management they influence the productivity of a wide range of agricultural practices. *Chlamydiae* are obligate intracellular bacteria that are known to be responsible for a wide range of serious global health-care challenges. From the *Chlamydiaceae* family within the *Chlamydiae* order, *Chlamydia trachomatis* and *Chlamydia pneumoniae* cause common human diseases, while *Chlamydia abortus*, *Chlamydia psittaci*, and *Chlamydia suis* represent zoonotic threats or are endemic in human food sources [1]. *C. trachomatis* is the leading cause of trachoma, which can lead to blindness if left untreated [2]. The same organism is also the most prevalent cause of sexually transmitted diseases worldwide [3]. *C. pneumoniae* has a 60%–70% seroprevalence in adults in western countries, and is associated with respiratory disease, pulmonary disease, atherosclerosis, and Alzheimer’s disease [4]. The majority of our knowledge and research efforts from the past 50 years has focused on *Chlamydiaceae* as the most widely known bacterial family of the *Chlamydiae* order. However, in the past 20 years there has been the addition of eight families of genetically related obligate intracellular bacteria. These new families are often collectively referred to as “*Chlamydia*-like organisms” (CLOs), “*Chlamydia*-related bacteria” or “environmental *Chlamydiae*” [1]. The identification of such a diverse range of new *Chlamydiae* has promoted renewed interest in understanding chlamydial bacteria and chlamydial disease management.

More reliable methods to identify chlamydial infections are highlighting that they may be far more prevalent and significant than previously identified [5,6]. Although chlamydial infections within the human population are currently manageable with existing conventional therapies [7,8], chlamydial infections within livestock are of significant concern [6,9]. Chlamydial infections are endemic in the livestock industry, and it has been shown that antibiotics typically only manage the severity of infection rather than achieve clearance on a herd level [9]. Reports of tetracycline-resistant *C. suis* in swine herds continue, and chlamydial persistence and/or reinfection are the norm in many herd settings [10]. Both symptomatic and asymptomatic persistence, in animals and humans, is a serious problem. It has been proposed that increasing treatment durations for chlamydial infections may resolve persistent infections [11], and prophylactic treatment for a wide range of pathogenic microorganisms is common in livestock management [12]. However, extended exposure of *Chlamydia* to antibiotics provides greater opportunity for the development of antibiotic resistance in chlamydial species as well as other pathogenic microorganisms [10]. Therefore, the practice of extended treatment durations to prevent, manage, or resolve chlamydial infections should be used with caution, particularly with regards to livestock at a herd level.

Many common chlamydial strains have shown the potential to develop varying degrees of resistance to standard antibiotics in in vitro settings [13]. However, based on recent follow-up studies of the community-wide treatment of common human chlamydial pathogens, it seems that the development of resistance to front-line antibiotics is unlikely [14,15]. Of greatest concern is the largely underestimated zoonotic potential of livestock chlamydial pathogens [9]. If poor livestock management practices continue, such as generalized prophylactic antibiotic regimes, we may eventually see the appearance and introduction of resistant chlamydial strains into human circulation [10]. Vaccines provide an attractive option for chlamydial management, however, there currently exists commerically available vaccines for only two chlamydial species: *C. abortus* in sheep and goats and *C. felis* in cats [9]. Although there is significant progress in the devlopment of various chlamydial vaccines, the complex biphasic chlamydial lifecycle and tendency for asymptomatic persistent infections provide significant challenges [16]. At this time, to better manage chlamydial infections in both humans and animals, it is important to continue the search for new antichlamydial compounds.

The aim of this review is to summarize the research exploring natural products as leads to developing novel antibacterial compounds for various *Chlamydiaceae* species, as the most widely studied family of the *Chlamydiae* order. An overview of *Chlamydiaceae* infections and treatment is provided, followed by a review of various biomedical phytochemical groups and their associated anti-infective action. Polyphenolic compounds are discussed, followed by lipidic compounds, proteinaceous compounds, cellular metabolites and probiotics, and several examples of polyherbal formulations which have shown to exhibit significant antichlamydial activity.

## 2. Overview of *Chlamydiaceae*

*Chlamydiales* is an order of bacteria and related prokaryotic organisms consisting of nine families. All species within the order can be identified by analysis of their 16S rRNA. The most widely known of these families is *Chlamydiaceae.* While some of these bacterial species are host-specific, others like *C. psittaci* and *C. abortus* represent a potential zoonotic threat [1].

### 2.1. Brief History

**Figure 1:**
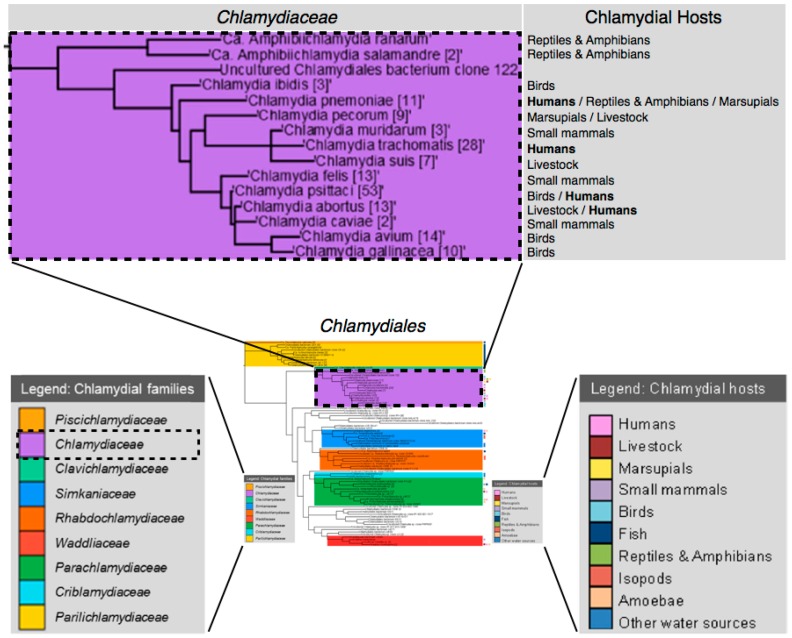
*Chlamydiaceae* species and hosts. From *Chlamydiales* phylogenetic tree with contents based on near full-length 16S rRNA gene sequences obtained from Genbank, NCBI. Adapted with permission from [1]. Copyright 2015 Oxford University Press.

In 1879, seven people in Switzerland were infected with *C. psittaci* by their imported tropical pet birds. This was the first discovery of *C. psittaci* as the cause of Psittacosis, or Parrot Fever. *C. trachomatis* was first discovered in 1907. However, both species were thought to be viruses until the first examination of the bacteria by microscopy in the 1960s. At the time, *C. psittaci* was thought to be part of the same genera as *C. trachomatis* and was named *C. psittaci*, while the strains found in animals were not known to be different species [17]. In 1985, Saikku et al., observed a connection between an unusual strain of *C. psittaci* and an epidemic of mild pneumonia in northern Finland [18]. This observation led to the identification of a new group of chlamydial organisms, which was termed TWAR in 1986 [19]. In 1988 and 1989, DNA–DNA hybridization studies of TWAR by Cox et al. [20] and Grayston et al. [21] revealed that it was a distinct chlamydial species, which became *C. pneumoniae* [22]. Work by Fukushi and Hirai in 1989 [23] led to the proposed existence of *Chlamydia pecorum* in 1992 [24]. In 1999, analysis of the 16S and 23S rRNA of *Chlamydiaceae* by Everett et al. indicated that there were two monophyletic lineages and nine species [25]. This was confirmed by other studies of 16S rRNA, the major outer membrane protein (MOMP), and ribosomal and coding genes. The other species of *Chlamydiaceae* were also discovered to be different species and named [26]. In 2000, *Chlamydiaceae* was defined as a family divided into two genera, *Chlamydia* and *Chlamydophila* [27], and comprised of nine species. However, in recent years, it has been proposed to shift back to a unification of the genera *Chlamydia* and *Chlamydophila* into a single genus [28]. In line with the majority of recent publications in the field, we are using the single genus “*Chlamydia”* in this paper. In 2014, two new species were identified [28], *Chlamydia avium* and *Chlamydia gallinacea*, and recent sources indicate that there are 11 *Chlamydia* species [1,29]. Although, research continues in this field and a 2015 review covering the past twenty years of research into chlamydia-like organisms identifies 15 *Chlamydia* species in the *Chlamydiaceae* family (Figure 1) [1].

### 2.2. Chlamydiaceae Infections and Treatment

**Figure 2:**
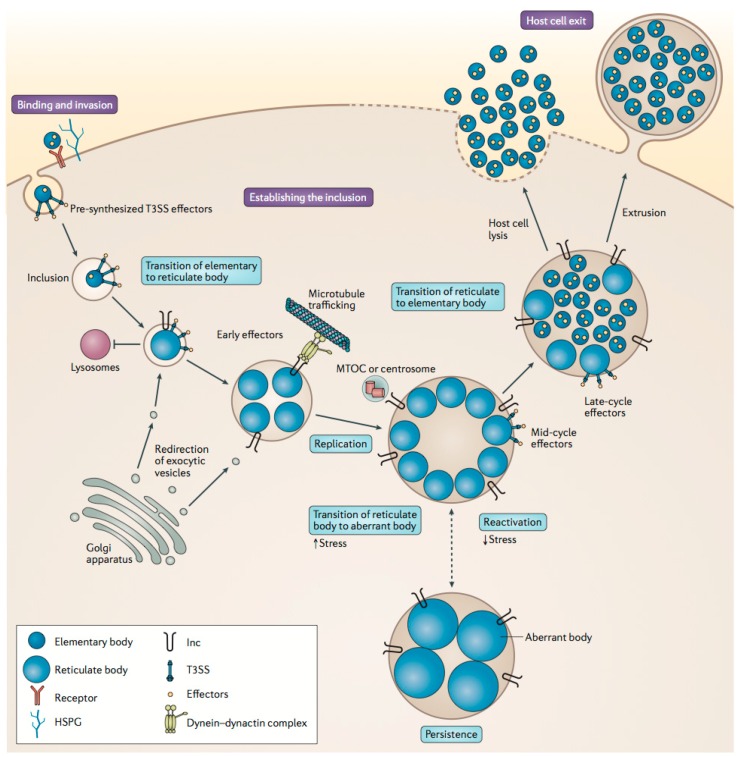
The life cycle of *Chlamydia trachomatis*. The elementary body (EB) binds to the host cell. Compounds are injected into the host cell to initiate internalization and establish an antiapoptotic state. The EB is incorporated into an endosomal membrane to form an inclusion, which is freed from the cell wall and passes into the host cell cytoplasm. Through bacterial protein synthesis the EB converts to reticulate bodies (RBs), which redirect host cell nutrients and divide by binary fission. The RBs direct host cell function and continue to replicate exponentially. If the RBs are excessively stressed they enter a dormant persistent state to promote survival, and reactivate upon removal of stress. Within 30–48 h the RBs differentiate back to EBs, which then exit the host cell through lysis or extrusion. Adapted with permission from [28]. Copyright 2016 Nature Publishing Group.

All *Chlamydiaceae* exhibit the same biphasic development cycle (Figure 2) where elementary bodies (EBs) enter host cells and transform into metabolically active reticulate bodies (RBs). Elementary bodies (EBs) traveling through extracellular secretions inside a host attach to a eukaryotic host cell and are taken in through phagocytosis. The EBs reorganize to form reticulate bodies (RBs), the replicating form of *Chlamydiaceae*. Reticulate bodies inhibit the formation of phagolysosomes and utilize host cell metabolic intermediates to counteract natural defenses. Inside an inclusion, or membrane-bound vacuole in host cytoplasm, RBs divide by asynchronous binary fission. After 30–48 h, the new RBs condense to form new EBs. The host cell then lyses, releasing the elementary bodies [29].

Although all *Chlamydiaceae* share a biphasic life cycle, there are differences between chlamydial species in standard treatment, potential for antibiotic resistance, and overall infection characteristics. Even within chlamydial species there exist serovar and strain variations which have shown to be more or less resilient to treatment [11]. Additional treatment variations can be required dependent on host, as well as the acute or chronic nature of infection. Overall, there exists significant complexity in the range of potential treatment requirements for chlamydial infections. However, Table 1 provides a broad summary of standard antibiotics for treatment of typical hosts, whether any in vitro or in vivo antibiotic resistance has been reported, whether asymptomatic persistence is common, and what range of tissues may be infected.

Frontline antibiotics for chlamydial infections include tetracyclines (TET) or macrolides (MAC), with doxycycline and azithromycin being the most common [5]. *C. trachomatis* within the human population remains manageable [7,8], although the potential for resistant forms has been reported [13]. Conversely, *C. suis*, which primarily infects swine, has shown to resist clearance at a herd level with a diverse range of antibiotics [12], and is the first of the *Chlamydiaceae* to develop stable TET-resistant forms in vivo [30]. *C. psittaci* responds well to TET or MAC treatment in humans and birds at an individual level [9,31], however, no reliable treatment is known for infections in cattle [6,32]. *C. pneumoniae* within the human population also remains manageable [5]. If detected and treated early, *C. abortus* in humans responds well to treatment with TET and erythromycin (MAC) [33], however, it is endemic in the livestock industry, and although oxytetracycline (TET) will reduce the number of abortions and bacterial shedding, antibiotics are ineffective in clearing the infection [9]. *C pecorum* is also endemic in the livestock industry [6], and in particular, no reliable treatment is known for infections in cattle [32], although, within Koala populations there has been some treatment success using chloramphenicol or enrofloxacin [34,35]. Other chlamydial species, such as *C. muridarum*, *C. felis*, and *C. caviae*, are generally manageable with TET and/or MAC treatment regimes [36,37,38]. *C. avium* and *C. gallinacea* have only recently been distinguished as distinct from *C. psittaci*, and for now it may be assumed that they have similar treatment and infection characteristics.

Livestock infections from *C. suis*, *C. psittaci*, *C. abortus*, and *C pecorum* all exhibit significant difficulty to clear, especially at a herd level [6,12,32]. Treatment failure, persistence, and reinfection are all common in a range of animal hosts, with antibiotics typically providing only a reduction in infection severity for the duration of the treatment period [6,10]. It is proposed that livestock often exhibit infection by multiple chlamydial species at a time [12,32], and chlamydial species found in livestock are commonly present in the digestive tract (Table 1), which may complicate clearance and allow for reinfection [39]. Recent research points to asymptomatic chlamydial infections being responsible for huge reductions in livestock productivity and profits [6]. There is a strong need for new treatment options to avoid widespread prophylactic antibiotic treatment in the industry as a means to effectively manage potential financial losses.

## 3. Biomedical Phytochemical Groups and Anti-Infective Action

Approximately 80% of the world relies on plant-based medicine for treatment of all ailments and 70% of pharmaceutical therapeutics used today are models of natural products [40]. Herbal medicines may be useful as a starting point for the development of new antichlamydial drugs [41]. Natural products comprise a diverse range of biochemical compounds. Plant secondary metabolites comprise the largest group of compounds used in plant-based therapeutics with polyphenols being the most commonly studied secondary metabolite group with respect to the treatment of *Chlamydiaceae* infections. Additionally, lipidic compounds and proteinaceous compounds are other commonly derived natural materials which exhibit antichlamydial activity. There have also been studies exploring the efficacy of cellular metabolites and probiotics to inhibit chlamydial infections. As examples highlighting the potential of natural product-based therapeutics, three broad-spectrum antimicrobial polyherbal formulations, which exhibit potent antichlamydial activity, have been developed and studied up to Stage II clinical trials.

### 3.1. Polyphenolic Compounds

**Figure 3:**
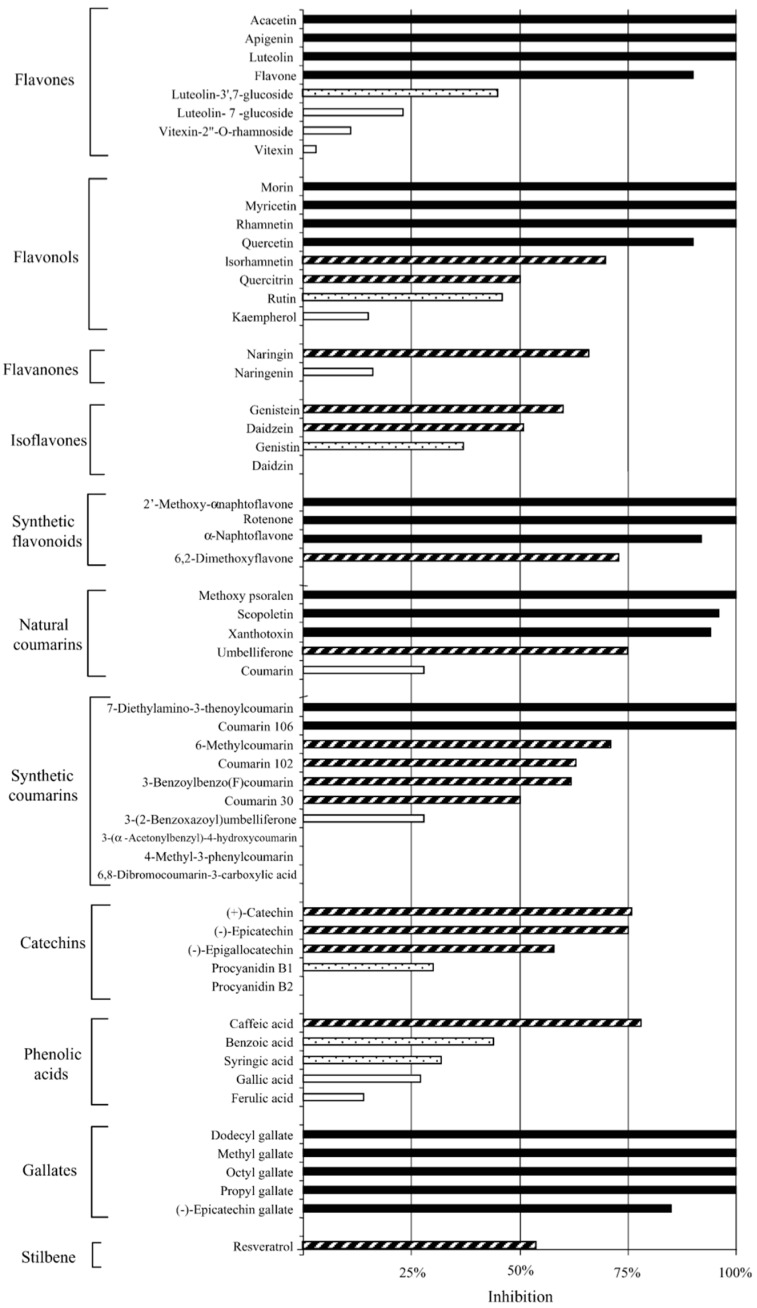
Inhibition percentages of various natural and synthetic polyphenolic compounds against *C. pneumoniae* at 50 μM concentration (*n* = 4 or more). Activity is determined in comparison to controls: highly active (black bar) = 85%–100% inhibition; active (striped bar) = 50%–84%; moderately active (black dotted bar) = 30%–49%; inactive (white bar) = <30%. Adapted with permission from [43]. Copyright 2005 Elsevier Inc.

Polyphenols are organic chemicals found in plants and have been shown to help prevent degenerative diseases and exhibit antimicrobial properties against a wide range of bacteria, including *Chlamydiaceae* [42]. Table 2 provides a summary of existing studies exploring the antichlamydial properties of polyphenols. The mechanism by which polyphenols have antichlamydial activity is not entirely known, however, based on these studies it appears that the antichlamydial activity of polyphenols is due to various modes of action. Results from a comprehensive study by Alvesalo et al. suggested that compound structural variations play a key role in the antichlamydial effect of polyphenolic compounds. This study evaluated the in vitro antichlamydial activity of 57 natural flavonoids and other natural polyphenols and structurally similar synthetic compounds against *C. pneumoniae* in human cells. Thirty-seven percent (21/57) of the studied compounds, all of which were non-toxic to the host cells at tested concentrations, were highly active against *C. pneumoniae*. From the remaining compounds, 28% (16/57) were considered active, 11% (6/57) moderately active, and 24% (14/57) inactive (Figure 3). Even when present only before infection, some compounds had the ability to accumulate inside cells or in cell membranes, causing inhibition of *C. pneumoniae* [43]. From this data, there are several observations of the relationship between molecular structure and observed antichlamydial activity. Compound structural variations, either free from sugar moieties, or with greater hydrophobicity, were found to be more active.

Early studies explored a group of polyphenols found in tea, known as catechins, which have been observed to exhibit broad-spectrum antimicrobial properties [44]. It has been proposed that catechins cause cytoplasmic membrane damage by damaging [45] or disrupting the permeability [46] of lipid bilayers. Yamazaki et al., performed an in vitro study on the effect of Polyphenon 70S, a tea polyphenol extract from a green tea high in catechins, on *C. pneumoniae-* and *C. trachomatis*-infected human cells. Complete inhibition of *C. pneumoniae* occurred at 1.6 mg/mL Polyphenon 70S for the strain AC-43 and at 0.8 mg/mL for the Ar-39 strain, and complete inhibition of *C. trachomatis* at 1.6 mg/mL for serovar D and at 0.4 mg/mL for the L2 strain. Polyphenon 70S comprises: epigallocatechin, epicatechin, epigallocatechin gallate, epicatechin gallate, and gallocatechin gallate. Epigallocatechin gallate is the dominant constituent and is attributed to be a major contributor to the observed antimicrobial effects. Although Polyphenon 70S was toxic to human cells at 0.25 mg/mL for treatment post-inoculation with the bacteria, it was non-toxic when treatment began pre-inoculation [47]. A further study by Yamazaki et al. showed that five biosynthesized tea polyphenols were active against *C. trachomatis* and analyzed the varying toxicity of the catechins. All five catechins, of which (−)-epicatechin (EC) was the least toxic, had an inhibitory effect on the proliferation of *C. trachomatis* in vitro. Because the concentration of tea polyphenols required for complete inhibition of *C. trachomatis* is high compared to antibiotics, tea polyphenols are not currently suitable for treating systemic infections. Modification of the catechin structure to reduce the required dose and toxicity may help circumvent the toxicity problem for use of catechins in a topical microbicide [48].

Other antichlamydial mechanisms have also been observed with several other polyphenols. Tormakangas et al., evaluated the effects of treatment of acute *C. pneumoniae* infection with the flavonoids quercetin and luteolin and an alkyl gallate, octyl gallate, in a mouse model. The lowest presence of *C. pneumoniae* in lung tissue was detected in mice treated with luteolin. The response to quercetin treatment was not favorable, which contradicts other studies performed with the flavonoid [43]. *C. pneumoniae* is reported to inhibit apoptosis of the infected host cell and it has been proposed that luteolin negates the antiapoptotic effect of chlamydia by inducing cellular apoptosis via interference with the mitochondrial pathway [49,50] and thereby allowing the chlamydial infection to become vulnerable to the hosts natural immune responses. The polyphenolic flavonoid, baicalin, also results in a more effective immune response to clear chlamydial infections. Baicalin is an antimicrobial, anti-inflammatory flavonoid isolated from *Scutellariae baicalensis*, or *Scutellariae radix,* a plant used in traditional oriental medicine and is a potential agent for therapy of *C. trachomatis* infections. Hao et al. found that baicalin can block the infection of Hep-2 cells. Treatment with baicalin began 24 days after inoculation with *C. trachomatis*. Some antichlamydial activity was observed with baicalin concentrations between 0.12 mg/mL and 0.48 mg/mL. At 48 mg/mL, baicalin strongly inhibited *C. trachomatis* and blocked further infection almost completely. When the RFX5 and chlamydial protease-like activity factor (CPAF) genes in chlamydia-infected cells were examined, it was found that RFX5 was upregulated and CPAF was downregulated by baicalin, with CPAF as the primary target [51]. It has been suggested that the CPAF degradation of RFX5 may play a role in chlamydia escaping efficient host immune detection, and therefore the down-regulation of CPAF may allow the hosts immune system to more effectively detect the chlamydial infection [41]. The lupine-class triterpene, betulin, is extensively distributed in nature and has been shown to be highly biologically active in the treatment of intracellular pathogens. Salin et al. extracted betulin from birch bark and evaluated thirty-two betulin derivatives for potential use against *C. pneumoniae* in vitro. Five promising compounds were identified. The betulin derivative, betulin dioxime, had a minimum inhibitory concentration (MIC) of 1 mM against the CWL-029 *C. pneumoniae* strain, with 50% inhibition achieved at 290 nM. A clinical isolate confirmed the antichlamydial activity, with an MIC of 2.2 mM. The mechanism by which inhibition occurs is unknown [52].

Studies on extracts from two different mint species showed positive antichlamydial effects. Corn mint (*Mentha arvensis*) contains various phenolic compounds, with rosmarinic acid, linarin, and acacetin compounds being most prevalent. Salin et al. examined the efficacy of corn mint extracts and several pure versions of compounds present in the corn mint extracts in treating *C. pneumoniae* infections both in vitro and in vivo. Both the mint extracts and pure compounds exhibited high levels of inhibitory activity in vitro, with low host cell toxicity. In a mouse model, intraperitoneally administered corn mint extracts delivered at nutritionally relevant concentrations resulted in reduced inflammatory symptoms but were not as effective as antibiotics in clearing the infection [53]. Kapp et al., performed an in vitro study on the effect of seven peppermint (*Mentha* × *piperita* L.) tea extracts on *C. pneumoniae*-infected human cells. While all seven tea extracts were active against *C. pneumoniae*, at an extract concentration of 250 μg/mL growth inhibition varied between 20.7% and 69.5%. Peppermint teas contain secondary metabolites, predominantly catechins and glycosides of flavanones and flavones, including luteolin and apigenin glycoside, which, along with rosmarinic acid, were related to higher antichlamydial activity [54].

Biochanin A is the main isoflavone component of red clover, and was compared with formononetin, genistein, daidzein, genistin, and daidzin for their antichlamydial effect against both *C. trachomatis* and *C. pneumoniae* in vitro. IC50 values ranged from 12 to >100 μM for all compounds, except formononetin and daidzein, which exhibited no inhibition of *C. trachomatis*. Overall, *C. pneumoniae* was more susceptible to inhibition. Hanski et al., determined that biochanin A exhibited the greatest antichlamydial activity overall, and significantly suppressed inclusion counts and decreased the mean bacterial inclusion size in *C. trachomatis*-infected cell cultures. Biochanin A prevented the formation of *C. pneumoniae* inclusions at concentrations of 25 μM or higher and prevented the formation of new infectious progeny. The greater efficacy of biochanin A was attributed to the presence of a methylated hydroxyl group. This study went on to improve bioavailability and the resulting efficacy of biochanin A with the development of oromucosal buccal dosage forms to improve dissolution and achieve permeation of buccal tissue so as to overcome digestive demethylation and conversion to genistein [55].

Polyphenolic synergistic effects with antibiotics or calcium modulators were explored and shown to result in improvements in antichlamydial efficacy. In an attempt to increase the efficacy of both polyphenols and doxycycline against *C. pneumoniae* in vitro, Salin et al. combined the polyphenols luteolin, quercetin, rhamnetin, and octyl gallate with either doxycycline or one of three calcium modulators—verapamil, isradipine, or thapsigargin. The polyphenol–doxycycline combinations did not increase the efficacy of treatment and some combinations had antagonistic effects. However, combining calcium modulators with polyphenols had some synergistic effect, although calcium modulators alone are not active against *C. pneumoniae*. While isradipine was synergistic with high concentrations of luteolin and quercetin, verapamil was synergistic with low concentrations of the same polyphenols. Thapsigargin had the greatest synergistic effect, significantly increasing the chlamydial growth inhibitory effect of polyphenols [56]. However, although this provides interesting insights, it should be highlighted that the synergistic use of calcium modulators in humans may lead to significant side effects. Rizzo et al. studied the synergistic effects of polyphenolic pretreatment followed by subsequent antibiotic treatment on *C. pneumoniae*. Either of the phenolic compounds resveratrol or quercetin was administered, followed by either clarithromycin or ofloxacin. With resveratrol concentrations of 40 μM and quercetin concentrations of 20 μM, both phenolic compounds exhibited significant inhibitory effects when combined with clarithromycin or ofloxacin, in comparison to controls. Chlamydial inhibition was linked to the immunomodulatory effects of decreased IL-17 and IL-23 production in a time-dependent manner in *C. pneumoniae*-infected cells [57]. Overall, the existing studies indicate that some polyphenolic compounds directly inhibit chlamydial activity by various mechanisms and may also work synergistically with other compounds to achieve increased efficacy.

### 3.2. Lipidic Compounds

**Figure 4:**
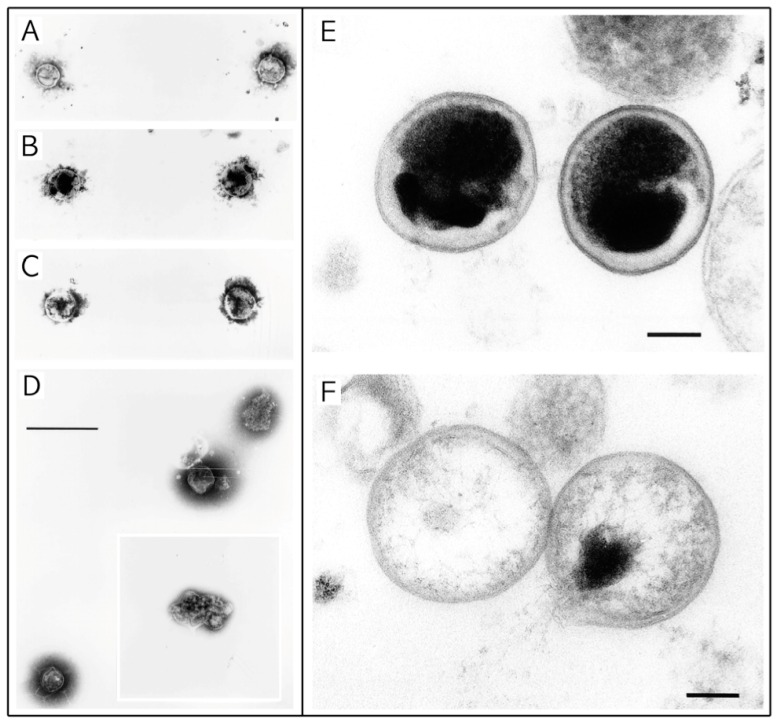
Scanning electron microscopy images of *C. trachomatis* with and without exposure to lipidic compounds. (**A**–**D**) Exposure of *C. trachomatis* EBs to monocaprin; The EBs were untreated (**A**) or treated with 10 mM monocaprin for 1 min (**B**); 5 min (**C**); and 10 min (**D**); With 10 min exposure the EBs appear deformed or disrupted (**D**, inset); Bars, 1 μm. (**E**,**F**) Exposure of *C. trachomatis* EBs to 1-*O*-hexyl-*sn*-glycerol; (**E**) *C. trachomatis* EBs exposed to sucrose-phosphate-glutamine buffer (SPG) only; (**F**) EBs exposed to 50 mM 1-*O*-hexyl-*sn*-glycerol for 90 min appear as hollow structures. Parts (**A**–**D**) are adapted with permission from [60]. Copyright 1998 American Society for Microbiology. Parts E and F are adapted with permission from [62]. Copyright 1998 American Society for Microbiology.

It has long been known that lipidic compounds, such as fatty acids, monoglycerides, and terpenoids, exhibit broad-spectrum antimicrobial effects [58]. Recent evidence supports that different kinds of lipidic compounds such as fatty acids and monoglycerides induce different kinds of morphological responses in lipid membranes, and hence are attractive to explore for therapeutic applications [59]. Table 3 provides a summary of existing studies that have explored the efficacy of lipidic compounds on chlamydial infections. Bergsson et al. evaluated the antichlamydial effects of 12 lipidic compounds on *C. trachomatis.* From these, monocaprin, lauric acid, and capric acid were shown to have the greatest antichlamydial effects. At a 5 mM concentration, the most active, monocaprin, caused a greater than 100,000-fold inhibition of *C. trachomatis* when exposed for five minutes. Capric acid was the least active of the three lipids, losing most of its activity when diluted. Monocaprin at 30 μg/mL was 50% effective when incubated with *C. trachomatis* for two hours. Chlamydial EBs were exposed to the lipid and then removed and inoculated into cell cultures. Observations of EBs exposed to monocaprin for 1, 5, and 10 min, indicated that after 10 min exposure, the EBs were irreversibly deactivated and were observed to rupture and disintegrate (Figure 4A–D) [60]. These observations support the proposal that lipidic compounds primarily inactivate the bacteria by affecting and disrupting the outer membrane.

Further studies went on to explore the potential of synthetic lipids for use in developing topical microbicides. Lampe et al. determined that 2-*O*-octyl-*sn*-glycerol, a synthetic lipid developed from naturally occurring human breast milk lipids was an effective inhibitory agent against *C. trachomatis*. Complete growth inhibition occurred after two hours of contact with a 7.5 mM concentration of 2-*O*-octyl-*sn*-glycerol. Four other lipids were studied, but were significantly less effective. When tested in conditions similar to the human vagina—10% human blood and with pH alterations between 4.0 and 8.0—lipid activity was not affected. When EBs were exposed to lipids for 90 min, the EBs appeared to be hollow shells with ruptured cell walls (Figure 4E,F). All of these lipids have also been shown to exhibit broad-spectrum antimicrobial properties [61], which supports cell wall disruption being the primary mechanism of action. However, it should be noted that these synthetic lipids were determined to exhibit no cellular toxicity, and preliminary observations indicate no vaginal irritation in rabbit models from lipid concentrations as high as 120 mM [62]. As a continuation of the work done in 1998 by Lampe et al., Skinner et al. explored the development of a topical microbicide with the synthetic lipid, 3-*O*-octyl-*sn*-glycerol [3-OG], and an engineered antimicrobial peptide, WLBU2, as the active compounds. After in vitro activity and toxicity analysis of the components, concentrations found to be toxic were omitted from further tests. While both WLBU2 and 3-OG were effective in vitro against *C. trachomatis,* the two components combined showed synergy, with significantly increased inhibitory activity. Although simulated fluids reduced activity, the combination shows potential for the development of a topical microbicide [63].

Another lipidic compound shown to have potential for topical application formulations is a tropolone-related compound, hinokitiol, which is found in the heartwood of trees in the *Cupresseceae* family. Hinokitiol has previously been shown to have antimicrobial activity against several bacterial species, including *Staphylococcus aureus* and *Schistosoma mansoni*. Yamano et al. studied the inhibitory effects of hinokitiol on *C. trachomatis*. Although, hinokitiol was found to be active against *C. trachomatis*, with an MIC of 32 μg/mL in vitro, the MIC was significantly greater than for hinokitiol against *S. aureus* or the MICs of antibiotics. Because high concentrations of hinokitiol are cytotoxic, topical application would be recommended for treatment of *C. trachomatis* infections [64]. While there are only a few studies exploring the antichlamydial efficacy of lipidic compounds, the results support the widely held opinion that lipidic compounds typically exhibit broad-spectrum antimicrobial properties, which is primarily due to disrupting the cellular membrane of pathogenic microbes. This supports the potential antichlamydial activity of a wide range of unexplored lipidic compounds, and highlights the need for ongoing research in this field.

### 3.3. Proteinaceous Compounds

There is a wide range of proteinaceous compounds known to exhibit antimicrobial effects. Table 4 summarizes existing studies reporting protein-based antichlamydial compounds. An early reference to the use of desert truffle (*Terfezia claveryi*) aqueous extracts in treating trachoma, a common eye disease resulting from a *C. trachomatis* infection, highlights the importance of exploring ethnobotanical therapeutics as leads for new antimicrobial compounds. Desert truffles are a mycorrhizal fungus, or dark brown truffle, native to the Arabian Peninsula, and are a traditional ethnobotanical medicine in Bahrain. A protein extracted from *T. claveryi* has displayed antimicrobial activity against a wide spectrum of bacteria and against *C. trachomatis* in particular. In a pilot clinical study conducted by Al-Marzooky in 1981, which used sterilized aqueous extracts of *T. claveryi* to treat patients with trachoma, the truffle extracts were found to be effective, although slower acting than conventional antibiotic treatment. The treatment that most effectively inhibited *C. trachomatis* was with partially purified proteins extracted from an aqueous extract of *T. claveryi*. Effective in treating a broad range of bacterial infections as well as other diseases, *T. claveryi* and other species of desert truffles have many potential uses in medicine in addition to treating *C. trachomatis* [65].

Peptides comprise short chains of amino acids and are a common form of antimicrobial proteinaceous compounds. There is significant research indicating the broad-spectrum antimicrobial properties of various peptide classes. During studies in the mid-1990s, Yasin et al. explored the antichlamydial properties of human defensin HNP-2 and porcine protegrin PG-1 against *C. trachomatis* in vitro. Both HNP-2 and PG-1 inhibited chlamydial infection, but HNP-2 was the most potent. Examination of PG-1 treated EBs revealed morphological changes, membrane damage, and loss of cytoplasmic contents [66]. Later, melittin, as a principle active component of bee venom, was explored by Lazarev et al. to develop a potential treatment for *C. trachomatis* infections. Chlamydial inhibition was achieved in vitro with the introduction and activation of recombinant plasmid vectors expressing the melittin gene. Melittin is known to be cytotoxic and it is believed that the main antichlamydial mechanism is its direct cytotoxic effect, however, a secondary mechanism may be due to lowering the transmembrane potential of a transfected cell, which disturbs chlamydial adhesion to the cell [67]. During in vivo mouse studies, half of infected mice were free from infection 3–4 weeks after exposure [68].

**Figure 5:**
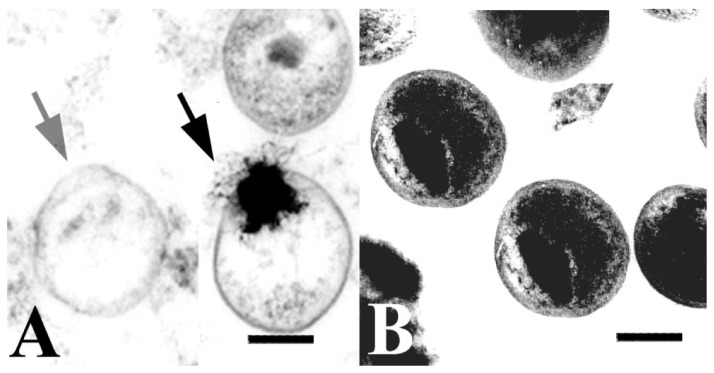
Scanning electron microscopy images of *C. trachomatis* exposed to cecropin peptide D2A21 for 90 min. (**A**) Organisms treated with D2A21 appear to be hollow or in the process of leaking their cytoplasmic contents (black arrow); (**B**) Untreated organisms incubated in SPG only. Bar = 0.5 μm. Adapted with permission from [69]. Copyright 2002 American Society for Microbiology.

Cecropin is a peptide found in cecropia moths (*Hyalophora cecropia*) that has been shown to have broad-spectrum antimicrobial activity. As part of the development of a cecropin-based topical microbicide, Ballweber et al. determined the minimum bactericidal concentration (MBC) of gel formulations of cecropin peptides D2A21 and D4E1 for in vitro activity against *C. trachomatis*. The gel formulations were equally effective against two *C. trachomatis* strains and the addition of 10% human blood did not alter the results significantly. Although pH values above and below 7 reduced D2A21 activity, the 2% D2A2 gel formulation remained effective with experimental pH variation. After D2A21 exposure for 90 min, ultrastructural observations showed that *C. trachomatis* EB membranes had been disrupted, causing the leaking of cytoplasm (Figure 5A,B) [69]. Previous studies have also suggested that this class of peptides inhibits microbial growth due to the creation of pores or channels through the bacterial membrane [70,71]. However, other studies suggest that similar peptides exhibit broad-spectrum microbial inhibitory activity due to the release of mitochondrial respiratory control, the inhibition of protein import, and the inhibition of bacterial respiration [72]. In an extension of the work done by Ballweber et al., as mentioned above, Skinner et al. explored the development of a topical microbicide with a synthetic lipid, 3-OG, and an engineered antimicrobial peptide, WLBU2. Both WLBU2 and 3-OG were effective in vitro against *C. trachomatis*, however, when combined, the two components together showed significantly increased inhibitory effect [63].

Granulocyte- and epithelium-derived antimicrobial peptides, protegrin-1, RTD-1, cryptdin-4, and indolicidin, were studied by Chong-Cerrillo et al. in vitro. Protegrin-1 was found to have the strongest antichlamydial activity, inhibiting infectivity by 50% at a concentration of 6 μg/mL. Several chlamydial serovars were examined and results suggested that specific peptide/bacteria interactions are complex and remarkably specific. Overall, it was observed that protegrins may have a broader antimicrobial activity than defensins [73]. Then, in a highly comprehensive study by Yasin et al., 48 structurally diverse antimicrobial peptides were examined against *C. trachomatis* serovar L2. By examining both natural and synthetic peptides from five major peptide groups: full-length β-sheet (×13), truncated protegrins (×7), PG-1 disulfide variants (×7), α-helical peptides (×12), and circular peptides (×6), it was possible to gain insight into some general properties regarding the antichlamydial properties of peptides. From this, it was proposed that moderate-sized cationic peptides may be useful in microbicide preparations designed to prevent chlamydial infection [74].

Cathelicidin peptides are found in lysosomes of macrophages and polymorphonuclear leukocytes (PMNs), and keratinocytes. In an important series of studies, Donati et al. explored the antichlamydial characteristics of cathelicidin peptides from various sources upon a wide range of chlamydial species. Initially, the cathelicidin peptides: SMAP-29 (sheep), LL-37 (humans), BMAP-27 (cattle), BMAP-28 (cattle), BAC-7 (cattle), and PG-1 (pigs), were tested against a total of 25 strains from the chlamydial species: *C. trachomatis*, *C. pneumoniae*, *C. felis*, *C. abortus*, *C. psittaci*, and *C. pecorum*. It was observed that: (1) SMAP-29 was most active against *C. trachomatis*, and was also active against *C. pneumoniae* and *C. felis*; (2) *C. pneumoniae* strains were less susceptible to peptides than *C. trachomatis*; (3) most animal *Chlamydiae* were not sensitive to cathelicidins at concentrations of around 10 to 80 μg/mL; and (4) PG-1 resulted in an increase in the number of inclusions in some animal chlamydial species at a concentration of 80 μg/mL [75]. In a follow-up study, the same group of peptides was examined against nine *C. suis* isolates obtained from pigs with conjunctivitis. Again, SMAP-29 was most active, followed by BAC-7 and BMAP-27, with LL-37 and PG-1 showing no activity at 80 μg/mL [76]. Next, to further explore the earlier observation that PG-1 resulted in increased activity in some animal chlamydial species, Donati et al. more carefully explored the effect of PG-1 on *C. abortus* infectivity. From this it was identified that infection of PG-1-pretreated cells resulted in an eight-fold increase in the number of inclusions and that PG-1 treatment after chlamydial infection had no increase in infectivity. Additional experiments demonstrated that PG-1 pretreatment facilitates the entry of *C. abortus* into host cells [77].

Dermaseptins are a family of peptides isolated from skin of the *Phyllomedusa* genus of frogs. Bergaoui et al. performed an in vitro evaluation of antichlamydial and cytotoxic properties of dermaseptin S_4_ and derivatives: D_4_D_20_S_4_, K_4_K_20_S_4_, S_4_ (5–28), and S_4_ (1–12). S_4_ provided 81% inhibition after 48 h at a concentration of 5 μg/mL, whereas K_4_K_20_S_4_ provided 96% inhibition after 48 h at 5 μg/mL. The 50% cytotoxic concentration (CC50) was determined to be higher than 25 μg/mL for each peptide, except for S_4_, which appeared to be more toxic than the other peptides. However, increasing the number of peptide positive charges reduced cytotoxicity [78]. Overall, there is a wealth of information regarding peptide/*Chlamydiae* interactions that could be used for the further development of peptide-based antichlamydial therapeutics.

Transferrins are a family of iron-binding glyco-proteins found in milk, tears, saliva, and vaginal secretions that have been shown to be both bactericidal and bacteriostatic [79]. A series of studies was performed to explore the efficacy of various transferrins on inhibiting poultry-related *C. psittaci* infections. Initially, Beeckman et al. tested the effect of ovotransferrin (ovoTF), human lactoferrin (hLF), and bovine lactoferrin (bLF) against *C. psittaci* in vitro. While all three transferrins exhibited inhibitory activity, ovoTF was more effective in inhibiting irreversible attachment and cell entry by *C. psittaci,* though transferrins had no effect on bacterial activity within eukaryotic cells. The antichlamydial activity of ovoTF is believed to stem from ovoTF incorporation into the bacterial membrane, followed by subsequent binding of bacterial lipopolysaccharides leading to the bacterial membrane becoming more rigid. It is proposed that increased membrane rigidity results in interference with the actin host cell recruitment pathway by disrupting the stability of bacterial T3SS translocon proteins, or by disrupting secretion of Type III secretion effector proteins. Additionally, interference with the actin host cell recruitment pathway may occur due to proteolytic degradation of the Type III secretion effector proteins. Such disruption of the actin host cell recruitment pathway is believed to inhibit actin host cell recruitment at the bacterial entry site, which is a key step in host cell internalization of the bacteria [80]. In a follow-up study, Van Droogenbroeck et al. investigated using ovoTF to prevent *C. psittaci* infections by treating turkeys with aerosolized ovoTF, and then inoculating the turkeys with a virulent strain of *C. psittaci*. While the ovoTF did not prevent infection, the severity of infection was significantly diminished. It is proposed that iron sequestration is involved in the in vivo antibacterial activity of ovoTFs and may also be the underlying mechanism which results in ovoTFs activating both innate and adaptive immune responses [81]. In a later study, newborn turkeys infected with *C. psittaci*, *avian metapneumovirus*, or *Ornithobacterium rhinotracheale* were treated with ovoTF. For the first nine weeks, the turkeys remained healthy, after which symptoms of respiratory disease appeared. OvoTF treatment reduced the shedding of *C. psittaci* into the air, reducing the risk of zoonotic transmission, and respiratory disease was delayed for the first half of the brood period, resulting in a 46% reduction in mortality. Although infection was not prevented, the symptoms of infection were less severe and the cost of antibiotics required for treatment lowered. As the *C. psittaci* infection was not cured, ovoTF is currently recommended for use in conjunction with antibiotics unless further studies increase the effectiveness of ovoTF [82]. These studies highlight the potential diversity in antimicrobial efficacy and mechanism of action for proteinaceous compounds. While transferrins can disrupt the biological pathways necessary for host cell infection, peptides have been shown to rupture the membrane of infective EBs thereby leading to bacterial lysis and death.

### 3.4. Cellular Metabolites & Probiotics

Mechanisms of immune response in controlling microbial infections and the complex interplay between symbiotic host microbiota and pathogenic microbes are important considerations in the development of new treatment strategies. Table 5 summarizes results of investigations into the effect of cellular metabolites and probiotic microbes with regards to chlamydial infections. Carratelli et al. explored the role of the cellular metabolite, nitric oxide (NO), in inhibiting *C. pneumoniae* from infecting macrophage J774 cells, as well as the ability of NO to directly damage isolated *C. pneumoniae* cells. Infected cells were exposed to recombinant murine gamma interferon (MurIFN-γ) so as to activate inducible nitric oxide synthase (iNOS). This resulted in increased production of NO and reduced viability of the infected cells. In addition, 2-(*N,N*-diethylamino)-diazenolase-2-oxide was added to cells before infection or during chlamydial cultivation. 2-(*N*,*N*-diethylamino)-diazenolase-2-oxide is a complex of diethylamine with NO, which can be used to generate a controlled release of NO in solution. The increase in NO concentration, before cell infection or during chlamydial cultivation, resulted in *C. pneumoniae* inhibition in a dose-dependent manner. These results suggest that the host immune response to chlamydial infection triggers cellular pathways which activate NO release and subsequently lead to the inhibition of chlamydial growth [83]. Cellular NO production also has implications for the role of symbiotic microbes in the control of pathogenic microbial infections, as a wide range of host symbiotic microbiota are known to produce NO as a cellular metabolite [84].

A growing trend in research is the role of symbiotic microbes in human health and results indicate that the host microbiome plays a significant role in the inhibition of pathogenic microbial infections. Pollmann et al. explored the efficacy of probiotic feed supplements to reduce rates of chlamydial congenital infections in swine. However, *C. suis* isolate, S-45, is identical to *C. trachomatis* serovar D, and consequently is a concern for zoonotic infection, such that limiting the spread of *C. suis* infection in swine is important for human safety. The large-scale antibiotic treatment of swine is undesirable, which has led to much interest in the development of alternative treatments. *Enterococcus faecium* is a bacterium that has previously shown beneficial effects as a probiotic feed supplement. When Pollmann et al. used *E. faecium* as a probiotic in the feed of pregnant *C. suis*-positive sows for thirteen weeks and then for eight weeks after giving birth, the rate of piglets born infected was reduced from 85% to 60% and the appearance of infection was delayed. The Pollman et al. study was the first to test the effect of probiotics on an obligate intracellular bacteria. It was proposed that *E. faecium* may function as an antibiotic by reducing the proliferation of bacteria, inhibiting host cell infection, or facilitating a more rapid clearance of the infection. Additionally, as a probiotic, *E. faecium* is expected to balance the native microbial population of the swine digestive tract, which is beneficial for maintaining the hosts innate defense system [85].

To gain greater insight into the mechanism by which probiotic bacteria may exhibit antichlamydial activity, there are several studies which explore the antichlamydial effect of various lactobacilli on *C. trachomatis*. Mastromarino et al. explored the antichlamydial effects of the vaginal lactobacilli, *Lactobacillus brevis* and *Lactobacillus salivarius*, on *C. trachomatis*. Both lactobacilli had an adverse effect on chlamydial EBs, on chlamydial adsorption to epithelial cells, and on intracellular phases of chlamydial replication, although *L. brevis* was significantly more effective than *L. salivarius*. Significantly, *L. brevis* inhibited the development of persistent forms of *C. trachomatis* induced by coinfection with herpes simplex virus type 2 (HSV-2) [86]. Gong et al. went on to explore the mechanism of lactobacilli antichlamydial properties on *C. trachomatis* by preparing and testing lactobacillus-conditioned media (LCM) from *Lactobacillus crispatus*, *Lactobacillus gasseri*, and *Lactobacillus jensenii*. The LCM from each of the lactobacillus strains exhibited similar inhibitory activity. Through pH analysis and modification of the LCM, chlamydial inhibition was shown to be due to acidic pH conditions arising from lactic acid production. As another cellular metabolite of interest, H_2_O_2_, was shown to not inhibit chlamydial activity at levels present in the LCM [87]. Rizzo et al., explored the ability of *L. crispatus* to influence the infectivity of *C. trachomatis*. HeLa and J774 cells were infected with *C. trachomatis* and exposed to *L. crispatus* and its supernatant. It was determined that *L. crispatus* and its supernatant had no cytotoxic effect on the epithelial cells or macrophages. Importantly, *L. crispatus* and its supernatant inhibited the adhesion of *C. trachomatis* cells to human epithelial cells or macrophages, and inhibited *C. trachomatis* infectivity. The immunomodulatory effect of *L. crispatus* was evaluated by variations in the expression of inflammatory cytokines, IL-6, IL-8, TNF-α, and IL-10. It was observed that *L. crispatus* and its supernatant reduced the production of the pro-inflammatory cytokines, IL-6, IL-8, and TNF-α. In contrast, *L. crispatus* and its supernatant significantly increased the production of the anti-inflammatory cytokine, IL-10. *L. crispatus* commonly resides in the urogenital microbiome of healthy women and these results suggest that increasing the presence of such microbes can play an important role in protecting the genitourinary tract against pathological conditions [88]. Nardini et al. performed a comprehensive study of eight strains of *L. crispatus*, six strains of *L. gasseri*, three strains of *Lactobacillus vaginalis*, and lactic acid as a lactobacilli cellular metabolite, on the infectivity of *C. trachomatis*. All lactobacilli exerted a strong inhibitory effect, although, *L. crispatus* exhibited the highest efficacy. Greater antichlamydial activity was correlated to increased cellular metabolites resulting in a lower pH, and the acidic conditions produced by lactic acid production were shown to be necessary for chlamydial inhibition. However, lactobacilli supernatants exhibited greater inhibition than only lactic acid, suggesting synergism with other lactobacilli metabolites. Interestingly, both shorter EB/lactobacilli supernatant incubation times, as well as increased lactobacilli consumption of glucose by the most active strains, were related to higher inhibitory activity [89]. Overall, these studies indicate the potential of a range of probiotics in inhibiting and managing chlamydial infections.

### 3.5. Polyherbal Formulations

**Figure 6:**
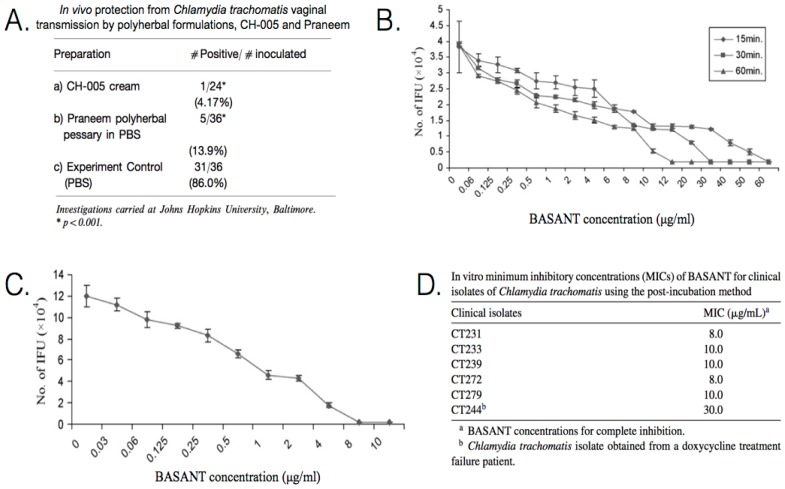
Efficacy of polyherbal formulations CH-005, Praneem, and BASANT. (**A**) In vivo protection from *Chlamydia trachomatis* vaginal transmission by polyherbal formulations CH-005 and Praneem; (**B**) Inhibitory effect of BASANT on *C. trachomatis* from pre-infection incubation. The number of inclusion-forming units (IFUs) decreases with incubation time and BASANT concentration; (**C**) Inhibitory effect of BASANT on *C. trachomatis* serovar D from post-infection incubation. The minimum inhibitory concentration (MIC) was determined to be 8 μg/mL BASANT; (**D**) in vitro MICs of BASANT for clinical isolates of *C. trachomatis* from post-incubation incubation. Standard deviations from triplicate tests are indicated by error bars. Part A is adapted with permission from [91]. Copyright 2000 American Journal of Reproductive Immunology. Parts (**B**–**D**) are adapted with permission from [93]. Copyright 2008 International Society of Chemotherapy.

There exist several polyherbal formulations which provide compelling insight into the antichlamydial potential of natural products. Table 6 summarizes the results of investigations into the effects of polyherbal formulations in the treatment of chlamydial infections. Praneem is a polyherbal formulation developed by Talwar et al. in the 1990s and has gone through Phase II trials for use as a spermicide and broad-spectrum antimicrobial. These studies have shown that Praneem is effective against *C. trachomatis*. The formulation has been used in the development of three antimicrobial products: a cream, a pessary, and an insertable tablet. Praneem contains saponins extracted from the pericarp of reetha tree (*Sapindus mukorossi*) fruit, quinine hydrochloride, and a purified extract from neem (*Azadirachta indica*) seeds as the main active ingredient. Toxicity studies indicate a lack of side effects, such as skin irritation or sensitization. Neem seeds contain water-soluble polysaccharides that stimulate antitumor and antiviral cytokines, including γ-interferon, which cause cell-mediated immune responses [90]. Praneem is more useful for preventing the spread of infections and treating some symptoms, as the polyherbal cream treats local, not systemic, infections [91,92]. In Phase I clinical trials, daily topical application of 5 mL of the cream for eight days, resulted in *C. trachomatis* being cleared from the cervicovaginal region of every subject studied [90]. The polyherbal formulation, CH-005, was also developed by Talwar et al. and studied alongside Praneem for its efficacy as a broad-spectrum antimicrobial against reproductive tract infections and sexually transmitted pathogens. The polyherbal formulation, CH-005, comprises purified saponins from *S. mukorossi*, *Mentha citrata* oil, and a natural polycationic polymer. In a mouse model study, topical application of either CH-005 or Praneem was effective in blocking the vaginal transmission of *C. trachomatis*, with CH-005 resulting in a 4.17% transmission rate and Praneem resulting in a 13.9% transmission rate (Figure 6A). It is claimed that additional studies, at John Hopkins University, to inhibit the vaginal transmission of *C. trachomatis* with a range of potential microbicides resulted in polyherbal formulations providing the best results [91].

BASANT is a polyherbal formulation developed by Bhengraj et al. that can be used as a cream or tablet as a vaginal microbicide, and the cream has gone through Phase II clinical trials in India. The incubation of cells with BASANT, both before inoculation with doxycycline-resistant *C. trachomatis* and after, showed antimicrobial activity against sexually transmitted serovars of *C. trachomatis.* BASANT is comprised of *Aloe vera*, curcumin, saponins from *S. mukerossi*, and amla (*Phyllanthus emblica*). It is proposed that the components have a synergistic effect that enhances the properties of each ingredient and broadens the spectrum of treatable infections. *A. vera* has wound healing properties and has been shown to inhibit HIV and HPV. Curcumin has antiseptic, anti-inflammatory, and antitumor properties. Amla has antioxidant, anti-inflammatory, and antimutagenic properties. In pre-infection in vitro studies, HeLa cells were infected with *C. trachomatis* serovar D and exposed to BASANT solutions of varying concentration. Based on pre-incubation exposure, complete inhibition was achieved with 15 min incubation at a concentration of 65 μg/mL, 30 min incubation at 35 μg/mL, and 60 min incubation at 15 μg/mL (Figure 6B). Based on in vitro post-incubation exposure, the minimum inhibitory concentration (MIC) was determined to be ~9 μg/mL (Figure 6C,D). With *C. trachomatis* isolates from a doxycycline treatment failure patient, the MIC was 30 μg/mL. There are no known side effects of BASANT, which is equally effective as a cream or tablet [93,94].

## 4. Conclusions

Through the utilization of various compound classes identified within these studies it seems likely that ongoing research in this field could lead to the development of effective antichlamydial products and treatment strategies. A wide range of natural compounds have been studied which exhibit varying degrees of antichlamydial activity. Polyphenolic compounds have been explored from commonly available sources, such as tea, mint, bark, clover, and traditional herbal therapeutics. Lipidic compounds have been explored in the form of purified fatty acids, breast milk lipids, and tree heartwood terpenoids. Proteinaceous compounds have been explored in the form of aqueous protein extracts from desert truffles, various peptides extracted from humans, pigs, sheep, cattle, moths, bee venom, and frog skin, and iron-binding glyco-proteins from humans, pigs, and chickens. Cellular metabolites & probiotics have been explored in the form of nitric oxide (NO), lactic acid, *E. faecium*, and six species of lactobacilli. Perhaps most importantly, three polyherbal formulations, utilizing various extracts from reetha tree fruit, cinchona bark, neem seeds, lemon mint, *Aloe vera*, turmeric, and Indian gooseberry, have demonstrated significant antichlamydial activity and highlight the potential for utilizing existing natural products to develop effective antichlamydial therapeutics.

To ensure long-term effective management of all *Chlamydiaceae*-related infections we should both prepare for the further development of clinical antibiotic resistance, as well as develop new options for providing better livestock management with the aim of preventing the development of antibiotic resistance. The exploration for new naturally derived or synthetic compounds is important, however, many modern antibiotics are already derivatives of natural products [95], and the further development of antibiotic resistance is always a possibility [96]. Although working with natural products is often challenging due to the biochemical complexity typically present, it is likely that this biochemical complexity offers a more reliable solution to infectious disease management.

Antibiotic resistance is the result of the natural evolutionary struggle between pathogens and hosts, and is often due to mutations in pathogenic bacteria, which eventually result in evolutionary solutions to improve on compounds with single mechanisms of action [96]. A standard approach to treating antibiotic resistant pathogens is multidrug therapy wherein two or more antibiotics are administered in an attempt to circumvent a pathogen’s ability to quickly adapt to a single stressor [97]. A multi-drug treatment may be more effective in treating chlamydial infections than a single drug approach, and it is important to devote greater effort into exploring synergistic antichlamydial compound combinations [98], as well as pursuing greater standardization of herbal therapeutics. Growing application of nanomedicine formulations for natural products in the context of antimicrobial applications of various kinds also deserves attention and could lead to improved formulations [99]. Overall, natural products show significant potential in treating chlamydial infections and the development of these products into novel drugs may help in the global management of *Chlamydiae*-related infections.

## Figures and Tables

**Table 1:** Standard antibiotic treatments, potential resistance, and infection characteristics.

Chlamydial Species	Standard Treatments	In Vitro and/or Natural Antibiotic Resistance [13]	Asymptomatic Persistence in Host [27]	Infected Tissues [27]
*C. trachomatis*	Humans [5] Doxycycline (TET) or azithromycin (MAC). Erythromycin ethyl-succinate (MAC), levofloxacin (FLQ), or ofloxacin (FLQ) are alternatives. Azithromycin (MAC) with amoxicillin (β-lac) or erythromycin (MAC) advised for pregnant women.	MAC, TET, β-lac, RIF, FLQ, SUL, TRI, LIN, AMI, FOS.	+	Eye, genital, joints, prostate, neonatal lung.
*C. suis*	Livestock (Swine) [12] NOTE: The following antibiotic treatments were unable to clear chlamydial infections on a herd level. Therapeutic treatment: aminoglycoside (AMI); β-lactam antibiotic (β-lac); cephalosporin (β-lac); fluoroquinolone (FLQ); or tetracycline (TET). Pro-/metaphylactic herd treatment: amoxicillin (β-lac); chlortetracycline (TET); MDT—chlortetracycline (TET), sulfadimidine (SUL), tylosin (MAC); or MDT—trimethoprim (DRI), sulfadimidine (SUL), sulfathiazole (SUL).	TET, β-lac, RIF, FLQ, SUL.	+	Eye, intestine, lung.
*C. muridarum*	Mice [36] Doxycycline (TET) or azithromycin (MAC).	TET, β-lac, RIF, FLQ.	+	Genital, intestine, liver, lung, kidney, spleen.
*C. psittaci*	Birds & Humans [9,31] Tetracyclines (TET) or macrolides (MAC). Erythromycin (MAC) is advised for children and pregnant women. Livestock (Cattle) [6,32] No evidence pointing to the successful use of antimicrobials to eliminate bovine chlamydial infection.	MAC, β-lac, RIF, SUL, TRI, AMI.	+	Brain, eye, genital, intestine, liver, lung, spleen.
*C. pneumonia*	Humans [5] Doxycycline (TET) or azithromycin (MAC). Clarithromycin (MAC), levofloxacin (FLQ), or moxifloxacin (FLQ) are alternatives. Azithromycin (MAC), clarithromycin (MAC), or erythromycin (MAC) is advised for children and pregnant women. 70%–86% efficacy with erythromycin (MAC), clarithromycin (MAC), azithromycin (MAC), levofloxacin (FLQ), or moxifloxacin (FLQ).	β-lac, RIF, FLQ, SUL.	+	Arteries, brain, joints, lung.
*C. abortus*	Humans [33] Responds well to early treatment with tetracyclines (TET) and erythromycin (MAC). Livestock (Ruminants) [9,10] Oxytetracycline (TET) will reduce the number of abortions and bacterial shedding. Antibiotics are ineffective in clearing the infection.	−	+	Intestine, placenta, spleen, fetal liver.
*C. pecorum*	Livestock (Cattle) [32] No evidence pointing to the successful use of antimicrobials to eliminate bovine chlamydial infection. Marsupials (Koalas) [34,35] Chloramphenicol (CHL) or enrofloxacin (FLQ) produce mixed results. Macrolides (MAC) and tetracyclines (TET) induce inappetence, emaciation, and death in koalas.	−	+	Bladder, brain, eye, intestine, lymph, joints, prostate.
*C. felis*	Cats [37] Doxycycline (TET) and topical tetracycline (TET). MDT: Amoxicillin/clavulanic acid (β-lac) may be a safe alternative for kittens.	−	−	Eye, genital, joints, lung.
*C. caviae*	Guinea Pigs [38] Doxycycline (TET) or azithromycin (MAC).	β-lac, RIF, FLQ, SUL.	−	Bladder, eye, genital, lung.
*C. avium*	Birds Tetracyclines (TET) or macrolides (MAC)—based on treatment for *C. psittaci*.	−	+	−
*C. gallinacea*	Birds Tetracyclines (TET) or macrolides (MAC)—based on treatment for *C. psittaci*.	−	+	−

TET: Tetracycline; MAC: Macrolide; FLQ: Fluoroquinolone; β-lac: β-lactams; AMI: Aminoglycoside; SUL: sulfonamide; CHL: Chloramphenicol; MDT: Multidrug therapy; + Asymptomatic persistance in host observed; − No supporting studies.

**Table 2:** Past studies exploring antichlamydial properties of polyphenolic compounds.

Antimicrobial Agent	Chlamydial Species	Study Design	Effects	Reference
Flavones (×8), Flavonols (×8), Flavonones (×2), Isoflavones (×4), Synthetic flavonoids (×4), Natural coumarins (×5), Synthetic courmarins (×10), Catechins (×5), Phenolic acids (×5), Gallates (×5), Stilbene (×1)	*C. pneumoniae* (K7)	In vitro Pre-inoculation: incubated with cells for 24 h prior to EB inoculation. Post-inoculation: administered at 0 h post inoculation (p.i.).	From 57 compounds, at 50 μM: 21 were highly active, 16 active, 6 moderately active, and 14 inactive. 10 compounds achieved an MIC of 50 μM or less with luteolin being 8.8 μM and dodecyl gallate being 18 μM. Gallates was the most active group. Some compounds accumulated inside cells or in cell membranes and cause inhibition when present only prior to infection. Compound structural variations, either free from sugar moieties, or with greater hydrophobicity, were found to be more active. All compounds were non-toxic to the host cells.	[43]
Polyphenon 70S: Epigallocatechin (18.3%), Epicatechin (8.6%), Epigallocatechin gallate (35.9%), Epicatechin gallate (11.2%), Gallocatechin gallate (3.5%)	*C. trachomatis* (D), (L2) *C. Pneumoniae* (AR-39), (AC-43)	In vitro Pre-treatment: incubated with EBs for 30, 60, or 90 min prior to inoculation. Post-inoculation: administered at 0 h p.i.	Polyphenon 70S, post-incubation, 100% inhibition of chlamydial inclusions, at 0.5 mg/mL, with toxicity to host cells at 0.25 mg/mL. Pre-incubation, 100% inhibition, at 0.4–1.6 mg/mL, with no toxicity to host cells.	[47]
Catechin, Epicatechin, Epigallocatechin, Epicatechin gallate, Epigallocatechin gallate	*C. pneumoniae* (AR-39)	In vitro Pre-treatment: incubated with EBs for 90 min prior to inoculation.	Inhibition was observed for concentrations from 0.4 to >6.4 mg/mL. Most active compounds were epigallocatechin gallate and epicatechin gallate, followed by epicatechin. Catechin and epigallocatechin exhibited intermediate activity. Epicatechin was the least toxic.	[48]
Luteolin, Octyl gallate, Quercetin	*C. pneumoniae* (K7)	In vivo Pre- & Post-inoculation: administered daily for 3 days prior to inoculation; administered daily for 12 days p.i. (mice)	Luteolin suppressed inflammation in lung tissue, *C. pneumoniae*-specific antibodies, and the presence of chlamydia in lung tissue. Octyl gallate had no significant effect on infection. Quercetin increased the inflammatory responses and the chlamydial load in the lungs.	[49]
Baicalin	*C. trachomatis* (D)	In vitro Post-inoculation: administered at 24 h p.i.	Blocks the infection of Hep-2 cells. Down-regulates the production of the chlamydia-secreted protein (CPAF). CPAF degradation of host transcription factors RFX5 may allow chlamydia to escape efficient immune detection. Baicalin may assist the host immune system to detect the chlamydial infection.	[51]
Betulin, Betulin derivatives (×32)	*C. pneumoniae* (CWL-029)	In vitro Post-inoculation: administered at 0 h p.i.	At a concentration of 1 μM, three derivatives showed >80% growth inhibition, and 15 compounds 20%–80% growth inhibition. Betulin dioxime exhibited an MIC of 1 μM, and achieved 50% inhibition at 290 nM. Compounds were well tolerated by host cells.	[52]
Corn mint extract (*Mentha arvensis*): Rosmarinic acid (5.2%), Linarin (6.0%), Acacetin-acetylglucoside-rhamnoglycoside (2.5%) Pure compounds: Rosmarinic acid, Linarin, Acacetin	*C. pneumoniae* (CWL-029), (K7)	In vitro Post-inoculation: administered at 0 h p.i. In vivo Pre- & Post-inoculation: administered daily for 3 days prior to inoculation; administered daily for 10 days p.i. (mice)	For corn mint extract, at 256 μg/mL, 73% inhibition of chlamydial inclusions was achieved for strain CWL-029, and 90% for strain K7, with ~78% host cell viability. Pure compound, inhibition at 100 μg/mL for strain CWL-029: linarin 100%, acacetin 97%, and rosmarinic acid 73%, with ~99% host cell viability. Pure compound, inhibition at 100 μg/mL for strain K7: linarin 62%, acacetin 81%, and rosmarinic acid 74%. In vivo, corn mint extract in nutritionally relevant dosages resulted in reduced inflammatory responses to chlamydial infection.	[53]
Peppermint tea extracts (*Mentha × piperita* L.): Eriocitrin, 12-Hydroxyjasmonate sulfate, Luteolin-*O*-rutinoside, Rosmarinic acid, Salvianolic acid B, Trace polyphenols, Trace plant acids	*C. pneumoniae* (K7)	In vitro Post-inoculation: administered at 0 h p.i.	Seven tea extracts were shown to be active against *C. pneumoniae*. At 250 μg/mL, from 20.7% to 69.5% inhibition. Higher content of luteolin and apigenin glycosides showed high activity. Host cell viability after the 72 h exposure to tea extracts ranged from 82.4% to 99.4%.	[54]
Isoflavones: Biochanin A, Formononetin, Genistein, Daidzein, Genistin, Daidzin	*C. trachomatis* (K), (L2) *C. Pneumoniae* (K7)	In vitro Post-inoculation: administered at 0 h p.i.	Biochanin A at 50 μM, complete inhibition of *C. pneumoniae*. Biochanin A, IC50: *C. trachomatis*—62 μM; *C. pneumoniae*—12 μM. No harmful effects on host cell viability. Biochanin A methylated hydroxyl group provides improved the antichlamydial activity. Biochanin A does not affect *C. pneumoniae* in its extracellular (EB) form. Oromucosal buccal dosage forms improve dissolution of biochanin A and allow for permeation of porcine buccal tissue.	[55]
Polyphenols: Quercetin, Luteolin, Rhamnetin, Octyl gallate Coadministrants: Doxycycline, Verapamil (Ca^2+^), Isradipine (Ca^2+^), Thapsigargin (Ca^2+^)	*C. pneumoniae* (CWL-029)	In vitro Post-inoculation: administered at 0 h p.i.	Quercetin, luteolin, rhamnetin and octyl gallate did not improve the antichlamydial effect of doxycycline. Some coadministration combinations of Ca^2+^ modulators with phenolic compounds resulted in potentiation of the antichlamydial effect of phenolic compounds. More antagonistic combinations were found than synergic or additive combinations.	[56]
Polyphenols: Resveratrol, Quercetin Coadministrants: Clarithromycin, Ofloxacin	*C. pneumoniae* (CWL-029)	In vitro Pre-inoculation: incubated with cells for 24 h prior to inoculation.	Resveratrol at 40 μM and quercetin at 20 μM exhibited significant growth inhibition in presence of clarithromycin or ofloxacin compared to controls. Immunomodulatory effects via strong inhibition of the IL-23 levels with coadministration of resveratrol or quercetin and ofloxacin or clarithromycin.	[57]

**Table 3:** Past studies exploring antichlamydial properties of lipidic compounds.

Antimicrobial Agent	Chlamydial Species	Study Design	Effects	Reference
Caprylic acid, Capric acid, Lauric acid, Myristic acid, Palmitoleic acid, Oleic acid, 1-monoglyceride of each fatty acid	C. trachomatis (K)	In vitro Pre-treatment: incubated with EBs for 1, 5, 10, or 120 min prior to inoculation.	Lauric acid, capric acid, and monocaprin caused >10,000-fold reduction in the infectivity titer. Monocaprin was the most active, with >100,000-fold inactivation of *C. trachomatis* at a concentration of 5 mM for 5 min. Results indicate that bacteria are killed by disrupting membranes of chlamydial elementary bodies.	[60]
1-*O*-octyl-, 2-*O*-octyl-, 1-*O*-heptyl-, 1-*O*-hexyl-, 2-*O*-hexyl-*sn*-glycerol	C. trachomatis (D), (F)	In vitro Pre-treatment: incubated with EBs for 0, 30, 60, 90, or 120 min prior to inoculation.	2-*O*-octyl-*sn*-glycerol, at 7.5 mM, completely prevented growth of *C. trachomatis* after 120 min of contact with the organism. The lipids were shown to have disrupted the chlamydial inner membrane, allowing leakage of the cytoplasmic contents from the cell.	[62]
Lipidic compound: 3-*O*-octyl-*sn*-glycerol Coadministrant: Antimicrobial peptide (WLBU2)	C. trachomatis (D), (E), (L2)	In vitro Pre-treatment: incubated with EBs for 5 or 120 min prior to inoculation.	3-*O*-octyl-*sn*-glycerol, at 6.25 mM, was 100% inhibitory after 5 min of exposure. Coadministration with WLBU2 produces significantly increased activity. 3-*O*-octyl-*sn*-glycerol could be used at up to 30 mM without causing toxicity.	[63]
Hinokitiol	C. trachomatis (D)	In vitro Pre-treatment: incubated with EBs for 1 h prior to inoculation. Post-inoculation: administered at 0 h p.i.	Hinokitiol, was shown to have an MIC and minimum lethal concentration (MLC) of 32 μg/mL. High concentrations of hinokitiol have been shown to be cytotoxic.	[64]

**Table 4:** Past studies exploring antichlamydial properties of proteinaceous compounds.

Antimicrobial Agent	Chlamydial Species	Study Design	Effects	Reference
Aqueous protein extract from mycorrhizal fungi (*Terfezia claveryi*)	*C. trachomatis*	In vivo Clinical treatment: administered to infected patients. (humans)	Sterilized aqueous *T. claveryi* extracts of were found to be effective, although slower acting than conventional antibiotic treatment. Partially purified proteins extracted from the aqueous *T. claveryi* extract were more effective.	[65]
Peptides: Human defensin HNP-2, Porcine protegrin PG-1	*C. trachomatis* (D), (H1), (L2)	In vitro Pre-treatment: incubated with EBs for 2 h prior to inoculation.	Both HNP-2 and PG-1 inhibited chlamydial infection, but HNP-2 was the most potent. PG-1-treated EBs exhibited morphological changes, membrane damage, and loss of cytoplasmic contents.	[66]
Peptide: Melittin	*C. trachomatis* (E)	In vitro Pre-treatment: incubated with EBs for 24 h prior to inoculation.	*C. trachomatis* inhibition after the introduction of recombinant plasmid vectors expressing the melittin gene. Main mechanism is its direct cytotoxic effect. Secondary mechanism is lowering the transmembrane potential of a transfected cell, which disturbs chlamydial adhesion to the cell.	[67]
Peptide: Melittin	*C. trachomatis* (D)	In vivo Pre- & Post-inoculation: administered 1 day prior to inoculation; administered at 14 days p.i.	Vaginal administration and induction of melittin gene transcription with doxycycline inhibited subsequent infection in mice. Half of the mice were free from infection within 3–4 weeks.	[68]
Peptides: Cecropin D2A21, Cecropin D4E1	*C. trachomatis* (D), (F)	In vitro Pre-treatment: incubated with EBs for 0, 5, 30, 60, 90, or 120 min prior to inoculation. Post-inoculation: administered at 0 h p.i.	D2A21, was shown to have a minimum cidal concentration (MCC) of 5 μM (18.32 μg/mL), and D4E1, an MCC of 7.5 μM (21.69 μg/mL). A 2% D2A21 gel formulation had an MCC of 0.2 mM (0.7 mg/mL). D2A21 incubation for 90 min caused chlamydial EB membranes to rupture causing the leaking of cytoplasm.	[69]
Peptide: WLBU2 Coadministrant: 3-*O*-octyl-*sn*-glycerol (3-OG)	*C. trachomatis* (D), (E), (L2)	In vitro Pre-treatment: incubated with EBs for 5 or 120 min prior to inoculation.	WLBU2, at 50 μM, was 89% inhibitory after 5 min of exposure, and 100% after 120 min. Coadministration with 3-OG produces significantly increased activity. WLBU2 could be used at up to 60 μM without causing toxicity.	[63]
Peptides: Protegrin-1, RTD-1, Cryptdin-4, Indolicidin	*C. trachomatis* (E), (L2), (MoPn)	In vitro Pre-treatment: incubated with EBs for 2 h prior to inoculation.	Protegrin-1 was found to have the strongest antichlamydial activity. Protegrin-1 inhibited the infectivity of the L2 serovar by 50% at a concentration (inhibitory concentration: IC50) of 6 μg/mL. Interaction between specific peptides and the various isolates tested appears to be complex and remarkably specific. Protegrins may have a broader antimicrobial activity than defensins.	[73]
Peptides: Full-length β-sheet (×13), Truncated protegrins (×7), PG-1 disulfide variants (×7), α-Helical peptides (×12), Circular peptides (×6)	*C. trachomatis* (D), (E), (L2)	In vitro Pre-treatment: incubated with EBs for 2 h prior to inoculation.	β-Sheet protegrins and α-helical peptides were equally active. Enantiomers were as active as native structures. Moderate-sized circular mini-defensins were less effective against *C. trachomatis*. Moderate-sized cationic peptides may be useful in microbicide preparations designed to prevent chlamydial infection.	[74]
Cathelicidin peptides: SMAP-29 (sheep), LL-37 (humans), BMAP-27 (cattle), BMAP-28 (cattle), BAC-7 (cattle), PG-1 (pigs)	*C. trachomatis* (A, D, E, H, I, LGV2) *C. pneumoniae* (IOL-207, CM-1) *C. felis* *C. abortus* *C. psittaci* *C. pecorum*	In vitro Pre-treatment: incubated with EBs for 2 h prior to inoculation.	SMAP-29 was most active, *C. trachomatis* inhibition by >50% at 10 μg/mL, with BMAP-27, BMAP-28, and BAC-7, >50% at 80 μg/mL. SMAP-29 also active against *C. pneumoniae* and *C. felis*. *C. pneumoniae* strains were less susceptible to peptides than *C. trachomatis*. Most animal chlamydiae were not sensitive to cathelicidins at concentrations of around 10–80 μg/mL. PG-1 at 80 μg/mL resulted in an increase in the number of inclusions in some animal chlamydial species.	[75]
Cathelicidin peptides: SMAP-29 (sheep), LL-37 (humans), BMAP-27 (cattle), BMAP-28 (cattle), BAC- 7 (cattle), PG-1 (pigs)	*C. suis* (MS04), (MS06 1–8)	In vitro Pre-treatment: incubated with EBs for 2 h prior to inoculation.	SMAP-29 was the most effective, six of the nine isolates, inhibition by >50% at 10 μg/mL (~3 μM). BAC-7 and BMAP-27, six of the nine isolates, inhibition by >50% at 80 μg/mL (~25 μM). LL-37 and PG-1 did not show any antichlamydial activity at 80 μg/mL.	[76]
Cathelicidin peptides: PG-1 (pigs)	*C. abortus* (S26/3)	In vitro Pre-treatment: incubated with EBs for 2 h prior to inoculation.	PG-1-pretreated cells, resulted in a ×8 increase in the number of inclusions. PG-1 treatment after chlamydial infection had no increase in infectivity. Experiments demonstrated that PG-1 pretreatment facilitates the entry of *C. abortus* into host cells.	[77]
Dermaseptin peptides: S_4_ D_4_D_20_S_4_ K_4_K_20_S_4_ S_4_ (5–28) S_4_ (1–12)	*C. trachomatis* (E)	In vitro Pre-treatment: incubated with EBs for 1 h prior to inoculation. Pre-inoculation: incubated with cells for 1 h prior to inoculation. Co-inoculation: inoculated cells simultaneously with EBs. Post-inoculation: administered at 0 h p.i.	S_4_, 81% inhibition after 48 h at 5 μg/mL. K_4_K_20_S_4_, 96% inhibition after 48 h at 5 μg/mL. 50% cytotoxic concentrations were determined to be higher than 25 μg/mL for each peptide, except for S_4_ at 10 μg/mL. Increasing the number of peptide positive charges reduced cytotoxicity.	[78]
Transferrins: Ovotransferrin, Human lactoferrin, Bovine lactoferrin	*C. psittaci* (D)	In vitro Pre-treatment: incubated with EBs for 1 h prior to inoculation. Post-inoculation: administered at 3 h p.i.	Ovotransferrin, pre-incubation, at 0.5–5 mg/mL, prior to infecting BGM cells significantly lowered infection rates. Ovotransferrin was more effective than human and bovine lactoferrin in inhibiting bacterial irreversible attachment and cell entry.	[80]
Transferrin: Ovotransferrin	*C. psittaci* (D)	In vivo Pre-inoculation: one dose administered pre-inoculation. Pre- & Post-inoculation: one dose administered pre-inoculation; administered daily for 12 days p.i. (turkeys)	A single pre-infection dose of 10 mg or a daily dose of 5 mg did not prevent turkeys from becoming infected with *C. psittaci*. Treatment significantly reduced the severity of infection.	[81]
Transferrin: Ovotransferrin	*C. psittaci* (D), (F), (E/B)	In vivo Prophylaxis: from 2 weeks old, administered daily for 12 days. (turkeys)	A daily 5 mg dose for 12 days prevented any symptoms of *C. psittaci* infection in turkeys. Respiratory disease occurred at 9 weeks although, overall treatment was associated with 46% reduction of mortality.	[82]

**Table 5:** Past studies exploring antichlamydial properties of cellular metabolites & probiotics.

Antimicrobial Agent	Chlamydial Species	Study Design	Effects	Reference
Nitric oxide	*C. pneumoniae*	In vitro Pre-treatment: incubated with EBs for 2 h prior to inoculation. Co-inoculation: inoculated cells simultaneously with EBs.	Increases in nitric oxide (NO) concentration resulted in chlamydial inhibition in a dose-dependent manner. Immune control of chlamydial infections may trigger NO production.	[83]
Enterococcus faecium	*C. suis*	In vivo Prophylaxis: from 24 days after mating, administered daily for 13 weeks for sows & 8 weeks for piglets. (pigs)	Swine consuming *E. faecium* for 13 weeks before and 8 weeks after giving birth, reduced the rate of infected piglets from 85% to 60%. The appearance of infection was also delayed.	[85]
*L. brevis*, *L. salivarius*	*C. trachomatis* (L2)	In vitro Pre-treatment: incubated with EBs for 1 h prior to inoculation. Co-inoculation: inoculated cells simultaneously with EBs for 1 h. Post-inoculation: administered at 0 h p.i.	*L. brevis* was significantly more effective than *L. salivarius*. Both lactobacilli had an adverse effect on chlamydial EBs, on chlamydial adsorption to epithelial cells, and on intracellular phases of chlamydial replication. *L. brevis* inhibited HSV-2-induced *C. trachomatis* persistence.	[86]
*L. crispatus* (×2), *L. gasseri*, *L. jensenii*	*C. trachomatis* (D), (L2)	In vitro Pre-treatment: incubated with EBs for 1 h prior to inoculation.	Lactobacillus-conditioned media from each of the lactobacillus strains exhibited similar inhibitory activity. Acidic pH due to lactic acid production was attributed to chlamydial inhibition. Levels of H_2_O_2_ present did not produce chlamydial inhibition.	[87]
*L. crispatus*	*C. trachomatis* (D)	In vitro Pre-inoculation: incubated with cells for 6 h prior to inoculation.	*L. crispatus* inhibits the adhesion of chlamydial cells to human epithelial cells or macrophages, and inhibited *C. trachomatis* infectivity. Modulation of inflammatory cytokines, IL-6, IL-8, and TNF-α, and anti-inflammatory cytokine, IL-10, was observed.	[88]
Bacteria *L. crispatus* (×8), *L. gasseri* (×6), *L. vaginalis* (×3) Cellular Metabolite Lactic acid	*C. trachomatis* (D)	In vitro Pre-treatment: incubated with EBs for 7, 15, or 60 min prior to inoculation.	*L. crispatus* exhibited highest efficacy although all lactobacilli exerted a strong inhibitory effect. Activity corresponds to increased cellular metabolites and a resulting lower pH. Both lactic acid and acidic conditions were necessary for inhibition. Lactobacilli supernatants exhibited greater inhibition than only lactic acid.	[89]

**Table 6:** Past studies exploring antichlamydial properties of polyherbal formulations.

Antimicrobial Agent	Chlamydial Species	Study Design	Effects	References
Praneem: *S. mukerossi* saponins, *A. indica* seed extract, Quinine hydrochloride	*C. trachomatis* (D)	In vivo Pre-inoculation: one dose administered prior to inoculation. (mice) Clinical treatment: administered daily for 7 days. (human)	Application of 5 mL of cream for 8 days, resulted in *C. trachomatis* being cleared from the cervicovaginal region of patients. Topical application is effective in blocking chlamydial vaginal transmission, with a transmission rate of only ~14%. Toxicity studies indicate a lack of side effects, such as skin irritation or sensitization.	[90,91,92]
CH-005: *S. mukerossi* saponins, *M. citrata* oil, Natural polycationic polymer	*C. trachomatis* (D)	In vivo Pre-inoculation: one dose administered prior to inoculation. (mice)	Topical application is effective in blocking chlamydial vaginal transmission with a transmission rate of only ~4%.	[91]
BASANT: *S. mukerossi* saponins, *A. vera*, *P. emblica*, curcumin	*C. trachomatis* (D)	In vitro Pre-treatment: incubated with EBs for 15, 30, or 60 min prior to inoculation. Post-inoculation: administered at 2 h p.i.	In vitro pre-incubation exposure, 100% inhibition was achieved in 15 min at 65 μg/mL, 30 min at 35 μg/mL, and 60 min at 15 μg/mL. In vitro post-incubation exposure, the MIC was determined to be ~9 μg/mL. There are no known side effects of BASANT, which is equally effective as a cream or tablet.	[93,94]
